# Inhibitory Control on a Stop Signal Task in Tourette Syndrome before and after Deep Brain Stimulation of the Internal Segment of the Globus Pallidus

**DOI:** 10.3390/brainsci11040461

**Published:** 2021-04-05

**Authors:** Francesca Morreale, Zinovia Kefalopoulou, Ludvic Zrinzo, Patricia Limousin, Eileen Joyce, Tom Foltynie, Marjan Jahanshahi

**Affiliations:** Department of Clinical and Movement Neurosciences, UCL Queen Square Institute of Neurology, 33 Queen Square, London WC1N 3BG, UK; francesca.morreale@hotmail.co.uk (F.M.); zinovia.kefalopoulou@hotmail.com (Z.K.); l.zrinzo@ucl.ac.uk (L.Z.); p.limousin@ucl.ac.uk (P.L.); e.joyce@ucl.ac.uk (E.J.); t.foltynie@ucl.ac.uk (T.F.)

**Keywords:** response inhibition, deep brain stimulation (DBS), Gilles de la Tourette’s syndrome, stop signal task, fronto-striato-subthalamic-palidal circuits

## Abstract

As part of the first randomized double-blind trial of deep brain stimulation (DBS) of the globus pallidus (GPi) in Tourette syndrome, we examined the effect of stimulation on response initiation and inhibition. A total of 14 patients with severe Tourette syndrome were recruited and tested on the stop signal task prior to and after GPi-DBS surgery and compared to eight age-matched healthy controls. Tics were significantly improved following GPi-DBS. The main measure of reactive inhibition, the stop signal reaction time did not change from before to after surgery and did not differ from that of healthy controls either before or after GPi-DBS surgery. This suggests that patients with Tourette syndrome have normal reactive inhibition which is not significantly altered by GPi-DBS.

## 1. Introduction

The ability to rapidly stop an action is an important function in everyday life. The neural substrates of such inhibitory control reside in the fronto–striato–subthalamic–pallidal circuits, which constitute a network for goal-directed and habitual inhibition of action, thoughts, memories and emotions [1].

Tourette syndrome (TS) is characterised by motor and/or vocal tics and attentional and psychiatric co-morbidities [2]. TS has been considered a basal ganglia disorder of inhibition, with tics resulting from increased activation of striatal neurons, causing inhibition of the globus pallidus (GPi) and substantia nigra pars reticulata (SNr) (which would normally be tonically active to prevent unwanted movements) and subsequent disinhibition of the thalamo–cortical targets. [3]. Imaging and neuropathology studies implicate cortico–basal ganglia circuits and abnormal distribution of inhibitory interneurons in the basal ganglia in TS [4,5,6]. Patients can temporarily suppress the tics, which suggests that they can impose goal-directed inhibition and intentionally suppress their involuntary tics, albeit for brief periods. It has been proposed that in TS tics may represent either overactivity in the generation of habitual actions or failure of automatic/habitual inhibition [1,7].

In recent years, the success of treatment of Parkinson’s disease and other movement disorders with deep brain stimulation has been extended to treatment of TS. In the first double-blind, randomized cross-over trial [8], it was established that deep brain stimulation (DBS) of the medial GPi is effective in controlling tics and some of the comorbidities of TS. In conjunction with this trial, we tested inhibitory control in TS patients on a stop signal task before and after GPi-DBS surgery to determine whether alteration of the output from the GPi, the final output pathway from the basal ganglia to the cortex, also alters inhibitory control on the stop signal task or not. 

## 2. Materials and Methods

### 2.1. Participants

Fourteen patients (11 male, mean age = 35.57, SD = 15.68 years), with severe TS were recruited from an ongoing randomised double-blind trial of bilateral GPi-DBS [8]. Informed consent was obtained. Most of the patients were receiving medications including antidepressants such as Selective Serotonin Reuptake Inhibitors and Serotonin Norepinephrine Reuptake Inhibitors, benzodiazepines, sleeping pills and/or antipsychotic medications. Due to psychiatric disturbance and difficulty in attending the clinic in one patient and the death of another patient unconnected to DBS surgery, only 12 of the 14 patients were assessed both before and after surgery. Details of the TS patients are presented in Table 1. Eight healthy volunteers (5 female, mean age = 33.13, SD = 6.79 years) also participated in the study. None of the healthy participants had any neurological conditions, psychiatric or physical illnesses or history of head injury or alcohol or drug abuse. 

### 2.2. Design and Procedure

A mixed between groups (TS vs. healthy controls) and within subjects (TS before and after GPi-DBS) design was used. Patients were tested on the stop signal task prior to surgery and for a second time when they had completed the trial and were confirmed to have GPi-DBS switched on. The healthy controls completed the stop signal task once. 

The study was approved by the local Ethics Committee. Informed consent was obtained from all participants. 

### 2.3. Stop Signal Task

The standard version of the Stop signal task was employed [9] involving three blocks of 96 Go trials (75% of all trials) and 32 Stop trials (25% of all trials). On the Go trials participants are required to respond to the presentation of a stimulus; either a left or right pointing green arrow. Participants were instructed to respond as quickly as possible using the index or middle finger of their dominant hand to the left or right pointing arrows, respectively. On the Stop trials a red cross was presented following the Go signal after a variable Stop signal delay (SSD) and participants had to stop themselves making the response. The inter-trial intervals randomly ranged from 0.5 to 4 s. Importantly, it was emphasised to participants that they should not allow their performance on the stopping task to interfere with their performance on the Go task. Participants were able to practise twenty trials before completion of the actual task. 

A staircase tracking procedure was used. For the Stop trials, the SSD values were sampled from one of four staircases, changing throughout the task based on the participant’s response. To begin with, the four staircases started with SSD values of 100, 150, 200 and 250 ms, respectively. Successful inhibition of a response on a Sop trial made inhibition more difficult on the next Stop trial by increasing the SSD by 50 ms. Conversely, if the participant failed to stop the response, then the SSD was reduced by 50 ms. Staircases of four step-up and step-down algorithms were used in this way to ensure convergence of P(inhibit) of 50% by the end of the three blocks. Go and Stop trials were mixed randomly. 

StopRespond reaction times (RTs) (RTs on stop trials on which participants failed to stop and responded) should be faster than Go RTs as the participant responds to the Go signal too quickly to allow inhibition following the Stop signal. The data were examined to identify any instances where the StopRespond RTs were longer than the GoRTs. 

Using the standard Race Model [9] the stop signal reaction time (SSRT) was estimated using an integration method calculated independently for each block, which overcomes problems associated with skewed GoRT distributions [10]. Go trials are ranked and the nth GoRT is obtained by multiplying the number of Go trials in the distribution by the probability of responding to the Stop signal. Finally, the mean SSD is subtracted from the nth GoRT to obtain the integrated SSRT value. The estimation of SSD was averaged from the mean values for the last six moves in each of the four staircases when the participant had converged on 50% inhibition.

Omission errors occurred on trials on which participants failed to respond to the go signal. Discrimination errors occurred on trials on which participants responded to the left or right arrow with the wrong finger. 

### 2.4. Statistical Analysis

Due to the relatively small sample sizes, non-parametric tests were used for most of the analyses. Mann–Whitney tests for between groups comparisons and Wilcoxon tests for the within-subject comparisons were employed. Effect sizes were reported with Cohen’s d. Pearson Correlation coefficients were also calculated. A Bonferroni correction was used to adjust the p value to control for the number of statistical comparisons made. The Bonferroni corrected *p* value was 0.002.

## 3. Results

The fourteen patients in the trial showed significant improvement of their tics with GPi-DBS (*t*(13) = 5.941, *p* < 0.002); see pre and post-DBS Yale Global Tic Severity Scale (YGTSS) in Table 1), an effect which was present in almost all cases [8]. There was also a significant improvement of quality of life (GTS-QOL) from before to after DBS-GPi surgery (*t*(11) = 4.092, *p* = 0.002). 

### 3.1. Comparison of TS before DBS Surgery with Age-Matched Healthy Controls

The patients and healthy controls were matched in terms of age (TS = 28.56, SD = 11.10; Controls = 33.13, SD = 6.79) (*t*(15) = −1.007, *p* = 0.330) and gender distribution (*X*^2^(1) = 3.085, *p* = 0.131).

**Figure 1:**
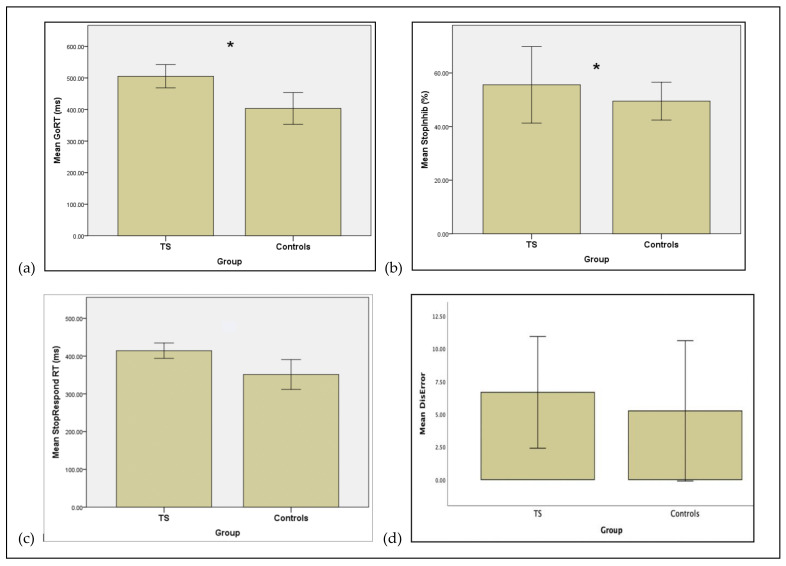
Mean measures for patients with Tourette syndrome (TS) **before** deep brain stimulation surgery and healthy controls. (**a**) Mean GoRT (ms) (**b**) Mean Stop Signal Delay (SSD, ms) (**c**) Mean StopRespond RT (StopRespond RT, ms). (**d**) Mean number of Discrimination Errors (Discrimination Error). Error bars represent standard errors of the mean. * Significant difference *p* < 0.002.

Both groups, achieved about 50% correct inhibition on the task, but the percent StopInhibit measure for the TS patients was significantly higher than for the healthy controls (*p* = 0.001). As shown in Table 1 and Figure 1a, the control group had significantly faster RTs (M = 403.50, SD = 70.89) to the Go signal than the TS patients (M = 505.35, SD = 55.41) (*U* = 11.00, *p* = 0.001).

Similar to the healthy controls, on the StopRespond trials on which participants failed to stop, the patient’s RTs were significantly faster than their Go RTs. This confirms an assumption of the race model. The StopRespond RTs for the control group were significantly faster than those of the patients (*U =* 12.00, *p* = 0.002) (Table 2, Figure 1c).

The average delay between the Go signal and the Stop signal, i.e., the SSD, was significantly longer for the patients compared to the controls (*U* = 10.00, *p* = 0.001) (See Table 1 and Figure 1b). The main measure of reactive inhibition, the SSRT for the TS patients before DBS surgery was not significantly different from that of the healthy controls (*p* = 0.071).

Patients made significantly more discrimination errors than the controls (*U* = 18.00, *p* = 0.012) (Table 2, Figure 1d), although this was not significant at the Bonferroni corrected *p* value. The two groups did not differ in terms of omission errors (*p* = 0.069).

The correlation of the SSRT with TS severity rating on the YGTSS was not significant before surgery (*r* = 0.58, *p* = 0.098).

### 3.2. Effect of DBS Surgery on Inhibitory Control on the Stop Signal Task—Within-Subjects Comparison

For within-subjects comparisons, Wilcoxon tests were completed to compare TS patient’s performance on the stop signal task before and after DBS-GPi surgery. 

TS patients achieved about 50% correct inhibition on the task both before and after surgery, and the difference in the percent StopInhibit measure was not different (*Z* = 1.051, *p* = 0.147). The average delay between the Go signal and the Stop signal, i.e., the SSD, was shorter for the patients after surgery, but the difference was not significant (*Z* = −0.943, *p* = 0.173) (Table 3). Neither of these changes were significant at the corrected *p* < 0.002 (Table 3). 

The patients Go RTs were faster after GPi-DBS than before (*Z =* −0.524, *p* = 0.3). The StopRespond RTs, trials on which patients failed to stop, were also faster after then before DBS-GPi surgery (*Z =* −0.943, *p* = 0.173) (Table 3). Again, neither of these changes were significant at the corrected *p* < 0.002.

The main measure of reactive inhibition, the SSRT was shorter after DBS-GPi surgery (*Z* = −0.734, *p* = 0.231) (Table 3), but the change was not significant at the Bonferroni corrected *p* < 0.002. 

### 3.3. Effect of DBS Surgery—Comparison of Post-DBS Data of TS Patients with Healthy Controls

A series of Mann–Whitney U tests were conducted to compare the performance of TS patients after GPi-DBS with healthy controls on the various measures of the Stop signal task. 

**Figure 2:**
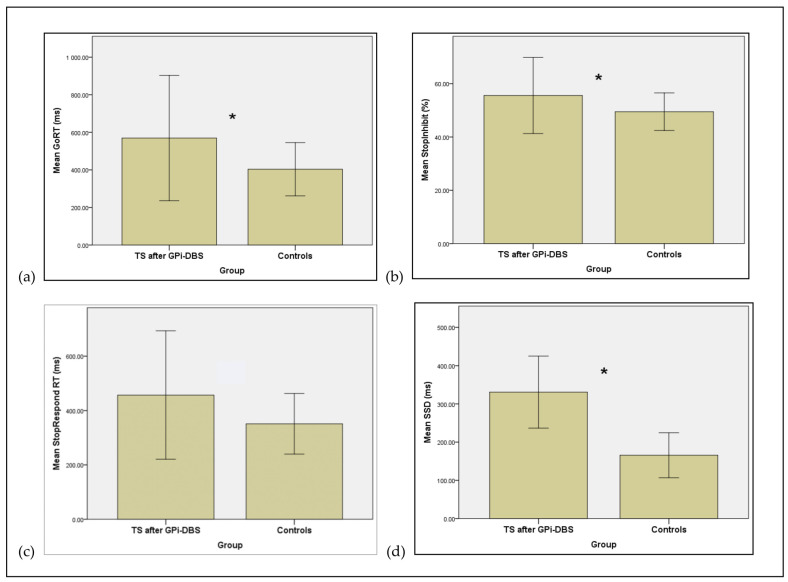
Mean measures for patients with Tourette syndrome (TS) **after** deep brain stimulation of the internal segment of the globus pallidum (GPi-DBS) and healthy controls. (**a**) Mean Go RT (ms) (**b**) Mean Stop Inhibition (percentage trials with correct inhibition) (**c**) Mean Stop Respond RT (StopRespond RT, ms) (**d**) Mean Stop Signal Delay (SSD, ms). Error bars represent standard errors of the mean. * Significant difference *p* < 0.002.

As shown in Table 4, the control group had significantly faster Go RTs (than the TS patients with GPi-DBS (*U* = 16.000, *p* = 0.010) (Table 4, Figure 2a).

While as a result of using the staircase procedure, both the patients and controls achieved correct inhibition on approximately 50% of the trials, the percentage of correctly inhibited responses following presentation of a Stop signal was higher for the TS patients after GPi-DBS than the healthy controls, with the difference being significant (*U* = 16.00, *p* = 0.001) (see Table 4, Figure 2b).

On Stop trials for which participants failed to stop, the patient’s StopRespond RTs were significantly faster than their GoRTs. The same was true also for the healthy control group’s StopRespond RTs. This confirms an assumption of the race model. The StopRespond RTs for the controls were significantly faster than those of the patients (*U* = 20.00, *p* = 0.004), albeit not at the BonFerroni corrected *p* value of *p* < 0.002 (see Table 4, Figure 2c).

The SSD was significantly longer for the TS patients compared to controls (*U* = 16.00, *p* = 0.001) (see Figure 2d and Table 4). The main measure of reactive inhibition, the SSRT, was not significantly different for the TS patients after GPi-DBS surgery and the healthy controls (Table 4).

The correlation of the SSRT after GPi-DBS with TS severity rating on the YGTSS after surgery was not significant (*r* = −0.15, *p* = 0.631).

## 4. Discussion

In TS, tics have been considered to reflect inhibitory dysfunction [1,3,7]. The aim of the current study was to evaluate inhibitory control in TS and the effect of GPi-DBS on response inhibition by testing a group of patients with TS before and after DBS of the GPi and comparing their performance to age-matched healthy controls on a task that involves motor inhibition: the stop signal RT task. Compared to healthy controls, the patients had significantly slower Go RTs and StopRespond RTs (trials on which Stop signal presented but participant failed to stop) both before and after GPi-DBS. Both before and after GPi-DBS, the main measure of reactive inhibition, the SSRT did not differ significantly for the TS patients from the healthy controls. Thus, none of the reaction time measures of inhibition were significantly altered by GPi-DBS surgery. 

### 4.1. Motor Inhibition in TS: Comparison of TS before DBS Surgery with Healthy Controls 

The current study found no significant differences in the measure of reactive inhibition on the Stop signal task, the SSRT, for the TS patients compared to healthy controls. This finding supports some previous studies of normality of inhibitory processing using the stop signal reaction time and other experimental tasks in TS [11,12,13,14,15,16,17,18,19,20,21]. 

A number of previous studies have reported enhanced inhibitory control in TS relative to controls [15,16,22]. It has been proposed that TS patients develop compensatory mechanisms in adolescence that allow the suppression of unwanted movements [16,22]. It is possible that TS patients were able to employ such compensatory mechanisms on the Stop signal task to inhibit responses when a stop signal was presented, which may account for the “enhanced” inhibitory control as revealed by the SSD and lack of impairment relative to healthy controls on the measure of reactive inhibition, the SSRT in our and other previous studies [11,18,19,20,21]. 

Recently, a review of the empirical evidence [1] distinguished between goal-directed and habitual/automatic inhibition mediated by fronto–striato–subthalamic–pallido–thalamo–cortical networks. The results of the imaging studies suggest that spontaneous tics are caused either by overactivity in the generation of habitual actions or by reduced activation of the mechanisms of habitual inhibition. As demonstrated in the present study of volitional motor inhibition, goal-directed inhibition can be engaged by individuals with TS. The main features of TS are consistent with faulty habitual or automatic inhibition [1,7], which results in the involuntary movements and utterances that constitute tics. Thus, while it was shown that volitional and goal-directed motor inhibition is not impaired in TS, and in fact some aspects were enhanced, the prediction would be that patients with TS would have deficits in automatic inhibition on tasks such as masked priming which involve automatic inhibitory processes. In fact, such a deficit in automatic habitual inhibition on a masked priming task and normal reactive and proactive inhibition on a conditional stop signal task has been recently demonstrated in TS [20]. 

### 4.2. Effect of GPi-DBS Surgery on Motor Inhibition in TS

Previously, a reduction in tics and notable clinical improvement in the same sample of TS patients following GPi-DBS surgery has been demonstrated [8]. For the first time, our study assessed the effect of GPi-DBS in TS on motor inhibition on the stop signal task. In the within-subject comparison, there were no significant differences in performance of TS patients after compared to before surgery, suggesting that stimulation of the GPi did not have an effect on reactive motor inhibition. Similarly, a comparison of TS patients before and after GPi-DBS with healthy controls also leads to a similar conclusion and highlighted that GPi-DBS did not produce any significant change in reaction time measures of reactive inhibition on the Stop signal task, other than a non-significant reduction in discrimination errors. 

### 4.3. Limitations of the Study

Certain limitations relating to the task and the sample size may have influenced the observed results. The sample size was small, particularly for the within-subject comparison of the effects of GPi-DBS. Due to our relatively small sample size, it was not possible to disentangle the contributions of the tic syndrome versus co-morbidities such as ADHD or OCD to inhibitory control in our study. In some previous studies [19,20,21], the influence of these comorbidities on performance of stop signal tasks has been examined. All three studies have consistently shown that while OCD is associated with longer SSRT and delayed reactive inhibition and also reduced grey matter volumes in a specific network [21], tic syndromes per se had no such effect on either SSRT or the imaging measures [19,20,21]. Other comorbidities such as ADHD, presence of depression or anxiety and psychotropic medication were shown to have no effect on measures of inhibitory control [20]. However, as previously noted [21], the specific characteristics of the sample such as age, specific comorbidities and the type of inhibitory task used may be critical in influencing inhibitory control in TS. The Stop signal task relies on externally generated actions that are stimulus driven and require goal-directed inhibition and therefore the SSRT provides a measure of reactive goal-directed inhibition. By contrast, these patients have motor and/or verbal tics that represent an impairment of habitual/automatic inhibition [1,7]. In fact, in support of this, while reactive and proactive inhibition on a conditional stop signal task have been shown to be normal in TS, habitual automatic inhibition on a masked priming task was impaired [20].

## 5. Conclusions

TS is considered a disorder of inhibition, relating to dysfunction of the cortico–striatal–thalamic–cortical pathways. The aim of the current study was to investigate reactive motor inhibition on the stop signal task in TS relative to healthy controls and the effect of GPi-DBS on response inhibition in TS. TS patients were assessed pre- and post-DBS surgery and results were analysed relative to healthy age-matched controls. The TS patients did not have an impairment of inhibitory control on the Stop signal task, as the SSRT measure of volitional reactive inhibition was not significantly different in TS relative to matched healthy controls either before or after GPi-DBS. 

## Figures and Tables

**Table 1:** Demographic and clinical characteristics of the patients with Tourette syndrome.

P	Sex	Age at Onset	Age at Surgery	Comorbidities	Stimulation Location	YGTSS Pre	YGTSS Post	GTS-QoL Pre	GTS-QoL Post
1	M	8	24	Anxiety	Posteroventral pallidum	87	63	82.4	74
2	M	11	22	Anxiety	Anteromedial pallidum	81	66	79.6	47.2
3	M	6	26	Obsessive Compulsive Behaviours (OCB)	Anteromedial pallidum	93	48	67.6	39.8
4	F	12	60	Depression	Anteromedial pallidum	93	49	62	14.8
5	M	7	25	OCB	Anteromedial pallidum	80	62	40.7	43.5
6	M	3	34	ADHD, OCD	GPi	93	58	N/A	18.5
7	M	6	21	Mild learning difficulties, traumatic myelopathy secondary to tics	GPi	94	83	78.7	47.2
8	F	10	25	Nil	GPi	94	37	63.9	28.7
9	M	6	39	Traumatic myelopathy secondary to tics	GPi	63	29	N/A	16.7
10	M	9	60	Segmental dystonia, probably secondary to neuroleptic use	GPi	60	22	42.6	41.7
11	M	6	18	OCD	GPi	94	51	80.6	47.2
12	M	7	32	OCD, Anxiety	GPi	74	74	81.5	82.4
13	F	9	55	Nil	GPi	93	4	47.2	1.9
14	M	-	38	OCD, Anxiety, Depression	GPi	82	47	52.8	48.2
**Mean (SD)**		**7.69 (2.46)**	**34.21 (14.5)**			**85.38 (11.69)**	**58.67 (21.16)**	**65.5 (15.94)**	**47.7 (22.2)**

YGTSS = Yale Global Tic Severity Scale; GTS-QOL = Gilles de la Tourette Syndrome Quality-of-Life Scale. ADHD=Attention Deficit Hyperactivity Disorder, OCD=Obsessive Compulsive Disorder.

**Table 2:** Means and standard deviations (in parentheses) of the measures on the stop signal task for patients with Tourette Syndrome (TS) **before** deep brain stimulation (DBS) surgery and healthy controls. RT = reaction time, SSD = stop signal delay, SSRT = stop signal reaction time.

Measure	TS Patients (*N* = 9) Mean (SD)	Controls (*N* = 8) Mean (SD)	*p*-Value (One-Tailed)	Effect Sizes (Cohen’s d)
GoRT ms	505.35 (55.41)	403.50 (70.89)	0.001	1.601
% StopInhibit	54.51 (3.08)	49.48 (3.52)	0.001	1.528
StopRespond RT ms	414.27 (30.45)	351.25 (55.76)	0.002	1.403
SSD ms	280.81 (69.37)	165.63 (83.16)	0.001	1.504
SSRT ms	224.54 (67.77)	237.87 (19.08)	0.071	0.268
Omission Errors	8.44 (23.48)	1.00 (1.41)	0.069	0.447
Discrimination Errors	6.67 (5.55)	5.25 (6.41)	0.012	0.237

**Table 3:** Means and standard deviations (in parentheses) of the measures on the stop signal task for patients with Tourette Syndrome (TS) **pre-DBS** surgery and **post-DBS** surgery. RT = reaction time, SSD = stop signal delay, SSRT = stop signal reaction time.

Measure	Pre DBS-GPi (*N* = 6) Mean (SD)	Post DBS-GPi (*N* = 6) Mean (SD)	*p*-Value (One-Tailed)	Effect Sizes (Cohen’s d)
GoRT ms	516.06 (61.00)	496.02 (56.13)	0.3	0.342
% StopInhibit	54.69 (2.61)	53.13 (2.55)	0.147	0.605
StopRespond RT ms	417.65 (33.28)	399.06 (36.11)	0.173	0.535
SSD ms	295.86 (65.73)	260.67 (70.73)	0.173	0.515
SSRT ms	220.19 (77.80)	235.35 (50.95)	0.231	0.231
Omission Errors	12.50 (28.68)	1.00 (1.55)	0.297	0.567
Discrimination Errors	6.5 (6.75)	5.50 (6.06)	0.342	0.156

**Table 4:** Means and standard deviations (in parentheses) of the measures of interest on the stop signal task for the patients with Tourette syndrome (TS) **after** deep brain stimulation of the internal segment of the globus pallidus (GPi-DBS) and healthy controls.

Measure	TS Post-Surgery (*N* = 11) Mean (SD)	Controls (*N* = 8) Mean (SD)	*p*-Value (One-Tailed)	Effect Sizes (Cohen’s d)
Go RT ms	569.60 (166.77)	403.50 (70.90)	0.001	1.296
% StopInhibit	55.59 (7.14)	49.48 (3.52)	0.001	1.09
StopRespond RT ms	456.79 (117.90)	351.25 (55.76)	0.004	1.144
SSD ms	330.89 (156.41)	165.63 (83.16)	0.001	1.319
SSRT ms	238.72 (51.25)	237.87 (19.08)	0.06	0.022
Omission Errors	0.91 (1.38)	1.00 (1.41)	0.06	0.065
Discrimination Errors	3.73 (4.82)	5.25 (6.41)	0.065	0.268
